# The Interplay between Child Maltreatment and Stressful Life Events during Adulthood and Cardiovascular Problems—A Representative Study

**DOI:** 10.3390/jcm10173937

**Published:** 2021-08-31

**Authors:** Vera Clemens, David Bürgin, Markus Huber-Lang, Paul L. Plener, Elmar Brähler, Jörg M. Fegert

**Affiliations:** 1 Department for Child and Adolescent Psychiatry/Psychotherapy, Steinhövelstraße 5, University of Ulm, 89075 Ulm, Germany; paul.plener@meduniwien.ac.at (P.L.P.); Joerg.Fegert@uniklinik-ulm.de (J.M.F.); 2 Department of Child and Adolescent Psychiatry, Psychiatric Hospitals of the University of Basel, Wilhelm Klein-Strasse 27, 4002 Basel, Switzerland; David.Buergin@upk.ch; 3 Institute of Clinical and Experimental Trauma Immunology, Ulm University Medical Centre, Helmholtzstraße 8/1, 89081 Ulm, Germany; Markus.Huber-Lang@uniklinik-ulm.de; 4 Department of Child and Adolescent Psychiatry, Medical University of Vienna, Währinger Gürtel 18-20, 1090 Vienna, Austria; 5 Department for Psychosomatic Medicine and Psychotherapy, University Medical Center of Johannes Gutenberg University of Mainz, Untere Zahlbacher Str. 8, 55131 Mainz, Germany; Elmar.Braehler@medizin.uni-leipzig.de; 6 Integrated Research and Treatment Center (IFB) Adiposity Diseases-Behavioral Medicine, Psychosomatic Medicine and Psychotherapy, University of Leipzig Medical Center, Philipp-Rosenthal-Str. 27, 04103 Leipzig, Germany; 7 Head of the Competence Area Mental Health Prevention Network Baden-Württemberg, Steinhövelstraße 5, 89075 Ulm, Germany

**Keywords:** child maltreatment, stressful life events, cardiovascular problems, obesity, diabetes, hypertension, myocardial infarction

## Abstract

Psychological stress is a major risk factor for cardiovascular diseases. While the relevance of early life stress, such as that which is due to child maltreatment (CM), is well known to impact individual stress responses in the long-term, and data on the interplay between CM and stressful events in adulthood on cardiovascular health are sparse. Here, we aimed to assess how stressful life events in adulthood are associated with cardiovascular health infarction in later life and whether this association is independent of CM. In a cross-sectional design, a probability sample of the German population above the age of 14 was drawn using different sampling steps. The final sample included 2510 persons (53.3% women, mean age: 48.4 years). Participants were asked about sociodemographic factors, adult life events, CM, and health conditions in adulthood. Results indicate that the number of experienced adverse life events in adulthood is associated with significantly increased odds for obesity (Odds Ration (OR)women = 1.6 [1.3; 2.0], ORmen = 1.4 [1.1; 1.9]), diabetes (ORwomen = 1.5 [1.1; 2.1], ORmen = 1.5 [1.1; 2.3]) and myocardial infarction (ORwomen = 2.1 [1.0; 4.3], ORmen = 1.8 [1.1; 2.8]). This association is not moderated by the experience of CM, which is associated with cardiovascular problems independently. Taken together, adult stressful life events and CM are significantly and independently associated with cardiovascular health in men and women in the German population in a dose-dependent manner. General practitioners, cardiologists and health policy-makers should be aware of this association between psychosocial stressors during childhood and adulthood and cardiovascular health.

## 1. Introduction

Psychological stress is a major risk factor for cardiovascular diseases [1]. Stress leads to an activation of the hypothalamus, and, consequently, to the activation of the two main stress systems of the human body: the hypothalamic–pituitary–adrenal (HPA) axis and the sympathetic nervous system (SNS). HPA axis activation results in cortisol secretion in the adrenal cortex and consequently increased intravascular volume and reduced inflammation, while the SNS leads to secretion of catecholamines in the adrenal medullar with consecutive increased heart rate, cytokines and reduced heart rate variability. Both HPA axis and SNS activation can lead to insulin resistance, increased blood pressure and increased coagulation [2].

One type of stress seems to be of particular relevance to health: stress during childhood, such as child maltreatment (CM). In the first large epidemiologic study assessing the impact of CM on physical health in adulthood more than 20 years ago, Felitti and colleagues found largely increased odds for several health problems including cardiovascular diseases in individuals who have experienced adverse childhood experiences (ACEs), encompassing CM and household dysfunctions, such as parental substance abuse or mental illness [3]. Today, there are numerous publications including cohort studies, adjusting for socioeconomic and psychosocial factors, showing the devastating impact of CM on adult health [4,5,6,7,8,9,10], leading to a reduction of lifespan up to 20 years [11].

CM is defined as “any act or series of acts of commission or omission by a parent or other caregiver that results in harm, potential for harm, or threat of harm to a child” [12]. Prevalence rates of CM are high. About one third of the German population reported at least one type of child maltreatment [13], comparable to global estimate rates [14]. Different subtypes of CM can be distinguished: emotional, physical, and sexual abuse, as well as emotional and physical neglect. In a representative sample of the German population, we have shown that the risk for obesity, diabetes, hypertension, and myocardial infarction is associated with each single subtype of CM and that growing intensity of each CM subtype is associated with higher rates of these conditions [15]. CM often co-occurs [16] and it seems to be particularly the accumulation of different types of CM leading to the devastating effects on long-term health [3,15,17,18,19].

Early life stress has been shown to affect long-term HPA axis functioning [20]. Consequently, cortisol metabolism [21] and autonomic, neuroendocrine, and inflammatory systems [22] are altered with potential long-term implications for hemodynamic and autonomic dysfunction [23]. This may be one explanation for why stress during childhood is associated with cardiovascular health impairments across the life span [22]. In a recent systematic review, CM was associated with cardiovascular diseases (myocardial infarction, stroke, ischemic heart disease, coronary heart disease) in 91.7% of the 24 included studies [24]. Yet, stressful life events do not stop when one reaches 18 years of age. Lifetime adversity, encompassing stressful events, such as loss of close persons, are associated with an increased risk for cardiovascular problems [25,26]. For stress before and after the age of 18, a dose-response relationship between the number of experienced stress types and cardiovascular risk factors and diseases was shown [3,15,17,18,19,27,28,29]. However, the interplay between CM and stressful life events in adulthood is not well explored. Altered cortisol responses to stress, such as after the experience of CM, have been shown to be associated with coronary artery calcification [30] and cardiovascular death [31]. As CM leads to long-lasting alterations in the individual stress response, including alterations in HPA-axis functioning and cortisol response to stressors [20,32] in a dose dependence manner [21], this may significantly impact the individual stress response to stressful life events in adulthood and consequently how the experience of stressful events in adulthood is associated with cardiovascular problems.

Therefore, in this study we first aimed to investigate how stressful life events in adulthood—specifically workplace mobbing, serious accident, loss of a partner or child, and sexual violence are associated with cardiovascular risk factors and myocardial infarction in later life. Secondly, we assessed whether the association between adult life events and later cardiovascular health is independent of CM.

## 2. Materials and Methods

A representative sample of the German population was generated in a three-step sampling approach by a commissioned independent research institute (USUMA, Berlin, Germany). Data collection took place between September and November 2016. Based on the municipal classification of the Federal Republic of Germany, in the first step systematic area sampling was used (ADM F2F Sampling Frame). Next, around 53,000 areas in Germany were delimited electronically, including an average of approximately 700 private households in each area. These areas were first layered regionally according to districts into a total of around 1500 regional layers and then divided into 128 “networks”. One network served as sampling frame, containing 258 single sample points proportionate to the distribution of private households in Germany. In the second step, private households were systematically selected with a random route procedure at each sample point. Households of every third residence in a randomly selected street were invited to participate in the study. As a last step, in multi-person households, a Kish selection grid was used to ensure random participation. To be included, participants had to be at least 14 years of age and required sufficient German language skills.

Individuals who agreed to participate in the study were given information about the study and provided informed consent. In the case of minors, participants gave informed assent with informed consent being provided by their caregivers. Participants were told that the study was about psychological health and well-being. Responses were anonymous. As the first step, research staff obtained socio-demographic information in an interview-format. Then, the researcher distributed a copy of the questionnaire and a sealable envelope. This was given back to the staff in the envelope after completion of the questionnaire. The completed questionnaires were linked to the respondent’s demographic data, but did not contain name, address, or any other identifying information.

Of 4902 designated addresses, 2510 households participated in the study (response rate: 51.2%). Main reasons for non-participation were refusal of the selected household to provide any information (15.3%), failure to contact any person in the household after four attempts (14.9%), refusal of the selected household member to participate (14.7%) and failure to contact the randomly selected household member after four attempts (2.3%).

The study was conducted in accordance with the Declaration of Helsinki and fulfilled the ethical guidelines of the International Code of Marketing and Social Research Practice of the International Chamber of Commerce and of the European Society of Opinion and Marketing Research. The study was approved by the Ethics Committee of the Medical Department of the University of Leipzig (protocol code: AZ 297/16-ek; date of approval: 13 September 2016).

### 2.1. Measures

Socio-demographic questions included age, gender, education, smoking and alcohol consumption.

The prevalence of child maltreatment was assessed using the Childhood Trauma Questionnaire (CTQ) [33,34,35]. The CTQ is a screening for the assessment of five subtypes of CM, sexual, emotional and physical abuse as well as emotional and physical neglect, assessed on five subscales. Psychometric properties of the German version of the CTQ reveal a high internal consistency ranging between 0.62 and 0.96 for all subscales [33]. Based on norm data by Haeuser and colleagues [36], severity scores for each subscale, ranging from “none-minimal”, “minimal-moderate”, “moderate-severe”, to “severe-extreme”, were calculated. In our analyses, dichotomous scores were used, based on scores reaching at least moderate-severe level. Based on this dichotomous scoring, the Cronbach’s alpha of the CTQ in our data set was 0.70.

To assess the number of stressful life events in adulthood, the participants were asked whether they ever had a serious accident at work, a car accident, or another serious accident, whether they ever had a spouse, life-partner, or child die, whether they ever have been forced to perform sexual acts against their will and whether they had ever been the victim of mobbing at their place of work. The sum of stressful life events that were experienced after the age of 18 was calculated.

Depressive symptoms were assessed with the Patient Health Questionnaire-2 (PHQ-2), a screening tool with a sensitivity of 82% and a specificity of 92% for major depressive disorder for a cut-point of ≥3 [37]. Anxiety was assessed with the Generalized Anxiety Disorder 2-item (GAD-2), a screening questionnaire with a sensitivity of 86% and a specificity of 83% for generalized anxiety disorder for a cut-point of ≥3 [38].

### 2.2. Statistical Analyses

All analyses were conducted using SPSS version 25. Descriptive analyses were used for prevalence, comparisons were performed using Chi-Square tests. Binary logistic regression analyses were then performed to identify predictors of cardiovascular problems. Age (in years), gender, smoking (yes/no), risk of alcohol abuse (yes/no), and educational level: achieved baccalaureate (yes/no) were entered in the analyses as co-variates.

In order to disentangle the interplay between CM and adult life stress for cardiovascular health, CM was then included into stepwise regression analyses in order to assess whether the association between adult life stress on cardiovascular health was independent of CM. As mental health is known to be associated with both, stress across lifespan [3,39] and cardiovascular heath [40,41], symptoms of anxiety and depression were included into analyses as potential confounder.

As there is evidence supporting that interpersonal victimization may have a more pronounced effect on health compared to other stressful live events [42], in an additional analysis, we have assessed the impact of interpersonal and other trauma separately. We have furthermore assessed forms of child abuse and child neglect separately using logistic regression analyses. Sexual abuse after the age of 18 years and workplace mobbing were categorized as interpersonal victimization while accidents and loss of a significant person were categorized as other adult stressful life events. Physical, emotional and sexual abuse were classified as “child abuse”, physical and emotional neglect as “child neglect”.

To confirm a potential moderation of CM in the association between adult life stress and cardiovascular stress, moderation analyses were conducted. Moderation analyses were performed with the PROCESS macro from Hayes [43]. Path analyses were performed with 5000 bootstrapping samples. The number of experienced forms of CM (0–5) served as a moderator variable.

## 3. Results

### 3.1. Sample Characteristics

In total, 2510 individuals (women: 53.3%, men: 46.7%) participated in the study. Any form of stressful life event after the age of 18 was reported by 678 participants (27.2%; women: 28.5%, men: 25.6). Child maltreatment was experienced by 744 participants (30.3%, women: 31.9%, men: 28.6%). In total, 416 participants (16.7%, women: 19.5%, men: 13.5%) reported obesity, 164 (6.6%, women: 6.4%, men: 6.8%) reported diabetes mellitus and 579 participants (23.3%, women: 23.8%, men: 22.7%) reported hypertension. In total 59 participants had experienced a myocardial infarction (2.4%, women: 1.1%, men: 3.9%) (see Table 1).

### 3.2. Adult Stressful Life Events Are Associated with an Increased Risk for Cardiovascular Risk Factors and Myocardial Infarction

**Figure 1:**
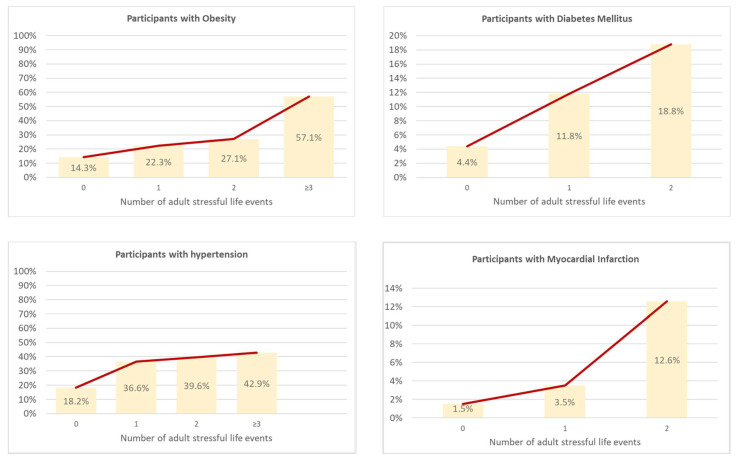
Cardiovascular problems and number of adult stressful life events. Analyzed via Chi^2^-Tests.

Nearly a quarter (22.3%, *n* = 127) of participants reporting stressful life events in adulthood were clinically obese, while this was only the case in 14.3% (*n* = 259) of those without stressful life events in adulthood (Chi^2^ = 35.97 (3), *p* < 0.001). Similar trends were observed in rates of diabetes (Chi^2^ = 62.52 (3), *p* = < 0.001), hypertension (Chi^2^ = 98.69 (3), *p* < 0.001), and myocardial infarction (Chi^2^ = 52.35 (3), *p* < 0.001; see Figure 1).

To exclude the possibility that these associations between the number of experienced stressful life events in adulthood and cardiovascular risk factors and myocardial infarction are driven by age or gender, we conducted binary regression analyses. Results revealed that with every additional stressful life event experienced after the age of 18, the odds for obesity increased about 50% in women (OR 1.61 [1.27; 2.04], *p* < 0.001) and men (OR 1.43, [1.06; 1.93], *p* = 0.019). The odds for diabetes increased by 52% [1.09; 2.13] in women (*p* = 0.014) and by 54% [1.05; 2.27] in men (*p* = 0.029). For myocardial infarction, with each additional adult stressful life event, the risk increased by 110% [1.03; 4.28] in women (*p* = 0.004) and by 76% [1.10; 2.82] in men (*p* = 0.019). After adjustment for relevant confounders, there was no significant association between the number of adult stressful life events and hypertension (see Table 2).

### 3.3. Adult Stressful Life Events and Child Maltreatment Independently Predict Cardiovascular Risk Factors and Myocardial Infarction

In the second step of our study, we aimed to assess whether the association between adult stressful life events and later cardiovascular health is independent of CM. After controlling for CM in regression analyses, the association between the number of stressful life events in adulthood and the odds for cardiovascular problems remained significant with no significant changes found in the OR. In detail, after controlling for CM, with each number of adult stressful life events, the odds for obesity increased by 37% [1.13; 1.66] fold (*p* = 0.002), the odds for diabetes by 37% [1.05; 1.78] (*p* = 0.019) and the odds for myocardial infarction 84% [1.23; 2.75] (*p* = 0.003). However, the condition of having also experienced CM was associated with significantly increased odds for cardiovascular health problems. With each additional category of CM, the odds for obesity increased by 11% [1.00; 1.23] (*p* = 0.041), the odds for diabetes by 21% [1.05; 1.40] (*p* = 0.008), the odds for hypertension by 15% [1.05; 1.27] (*p* = 0.005) and the odds for myocardial infarction by 38% [1.11; 1.71] (*p* = 0.004). Similarly, as with results calculated without controlling for CM, there was no significant association between the number of experienced stressful life events in adulthood and hypertension (see Table 3). Moderation analyses revealed no significant moderation of CM and adult live-events predicting and cardiovascular problems (see Table S1, Supplement).

### 3.4. Role of Interpersonal Victimization and Other Adult Stressful Life Events and Child Abuse and Neglect

In an additional analysis, we aimed to assess whether there is a difference between interpersonal victimization and other forms of adult stressful life events as well as between child abuse and neglect on cardiovascular health in adulthood. While interpersonal victimization was associated significantly with a higher risk for obesity (OR 2.54 [1.80; 3.59], *p* < 0.001) and hypertension (OR 1.53 [1.06; 2.21] *p* = 0.023), other stressful life events were associated significantly with a higher risk for obesity (OR 1.29 [1.03; 1.62], *p* = 0.026), diabetes (OR 1.69 [1.26; 2.26], *p* < 0.001) and MI (OR 2.04 [1.33; 3.15], *p* = 0.001). Regarding child abuse and neglect, child abuse was associated significantly with increased risks for obesity (OR 1.31 [1.08; 1.58], *p* = 0.006) and diabetes (OR 1.45 [1.11; 1.89], *p* = 0.006) while child neglect was associated significantly with an increased risk for hypertension (OR 1.22 [1.04; 1.44], *p* = 0.018) and MI (OR 1.52 [1.03; 2.25], *p* < 0.035) (see Table S2, Supplement).

## 4. Discussion

With this study, we were able to demonstrate for the first time that stressful life events in adulthood are associated with an increased risk for cardiovascular risk factors and myocardial infarction independently of CM in a population-representative sample.

Stress is known as a significant risk factor for cardiovascular problems [1,26]. Our results underline the association between stress and cardiovascular problems in a population representative sample. The findings are in line with literature, emphasizing the role of stressful life events as important contributors to cardiovascular disorders. In our sample, the association between stressful life events and cardiovascular problems was comparable in female and male participants. This is supported by other studies showing a significant effect of stress on cardiovascular health in both men and women [44].

Chronic stress exposure such as stress at work, loneliness and social isolation is well known to impact cardiovascular health [1,26]. However, there are studies showing the impact of stressful life events such as the death of a significant person in one’s life in large multicenter or nationwide studies [25,45]. Data focusing on other stressful life events is less available; however evidence exists, showing increased risks for cardiovascular problems after car accidents [46] and a growing number of studies demonstrates the relevance of bullying in adulthood for cardiovascular problems [47,48,49]. Similarly, evidence on the relevance of sexual assault as a risk factor for cardiovascular problems is growing [50,51,52]. Our data support the role of these stressful events as significant contributors to cardiovascular risk factors and MI in a large-scale representative sample of an industrial nation (Germany). Moreover, our data show that the accumulation of stressful events in adulthood are of particular importance for cardiovascular problems, pointing towards a dose-response relationship. This is well known for adverse childhood experiences (ACEs), where the number of experienced types of different ACEs are of major importance for physical and mental health in adulthood [3,10,15,17,18,19]. The same effects were shown before for the number of experienced stressful life events across lifespan and cardiovascular health [27,28,29]. Our data extends these findings by differentiating between stressful life events before and after the age of 18, showing that both types are significantly and independently associated with cardiovascular health.

No clear trend could be seen in our data between the relevance of interpersonal victimization and other stressful life events in adulthood. Our results are surprising as interpersonal victimization often goes along with chronic and more complex traumatization, leading to more complex impairments of mental and somatic health [42,53,54,55,56]. However the number of interpersonal victimization and other types of trauma was very limited in our study and important factors such as chronicity of the trauma was not assessed, which impaired the validity of our results.

Focusing on child abuse and child neglect were both relevant for cardiovascular health in adulthood, which is in line with literature, suggesting that the aftermath of neglect, particularly emotional neglect can be as devastating for health as the consequences of child abuse [57,58].

The effect size in our study was moderate, as the risk for cardiovascular problems increased by 20 to 50% with each additional stressful life event. These numbers are higher than in the study by Berntson and colleagues in a representative sample of the U.S. population, where an increase of risk for cardiovascular disease by 15% for each additional stressful life event was seen [27]. However, in the study by Berntson and colleagues, a greater number of stressful life events was assessed, such as problems with a neighbor, friend, or move. This may have impacted personal perceived stress less than the events in our study. The role of CM as a risk factor for cardiovascular health has been shown repeatedly before [3,10,15,17,18,19], including in the sample to which this study refers [15]. Besides biological mechanisms such as HPA axis dysregulation [59] and chronic inflammatory processes, encompassing increased levels of pro-inflammatory cytokines and oxidative stress [60,61], socioeconomic and behavioral aspects are discussed to mediate the association between stress during childhood and adulthood and health conditions in adulthood. These mechanisms encompass behavioral factors such as substance abuse, risky sexual behavior [4,62,63], sleeping problems [64] and smoking [65,66]. Further socioeconomic aspects comprise impaired social networks and relationships [67,68], lower academic achievements [69,70] and also lower socio-economic status [71,72,73]. Furthermore, chronic stress exposure during childhood and adulthood increases the risk for psychiatric disorders [4,74,75], which again may interact with cardiovascular health. Particularly depression is a well-known consequence of CM and often co-occurs with cardiovascular problems. As depression furthermore is associated with the above named behavioral and socioeconomic factors that are known to contribute to the development of cardiovascular disease, depression is discussed to mediate the shown association between stress and cardiovascular health [76]. However, in our analyses, the relationship between stressful life events and cardiovascular problems remained significant.

Of specific note, our results point towards a dose-response relationship between stressful life events before and after the age of 18 and cardiovascular problems—rather than towards a moderating role of CM in the association between stressful life events in adulthood and cardiovascular health. This finding is surprising, given the established long-term consequences of CM on the individual response to stressors in adulthood [20,22,32]. One explanation for why we have not found any moderating effect of CM may be that CM is not the only determinant for stress responses. Genetic factors, such as FK506 binding protein 5 (FKBP5) and G-protein coupled type-I CRH receptor (CRHR1), strongly influence individual stress reactivity [77]. The relevance of epigenetic modifications onto stress response and the development of health problems after stressful events has become more evident in studies during the last decade, particularly in stress-axis-, immune- and transmitter- related genes [78,79]. Further determinants encompass cognitive appraisal [80] and psychological coping resources [26]. Future studies should include measures of biological stress responses in order to disentangle the relevance of early life stress and stress in adulthood. The inclusion of genetic, epigenetic, and behavioral factors into studies with prospective cohort designs may give a more detailed answer for the questions regarding how an individual’s CM affects HPA-axis functioning over the long-term, how this long-lasting change may alter the individual stress response to stressful life events in adulthood, and consequently the stress-associated risk for cardiovascular problems.

While the population representative sample, allowing a control for major sociodemographic confounders and ensuring a high generalizability of the results, and the high response rate are major strengths of our study, its cross-sectional character is a major limitation. Chronological order of events and causality cannot be deduced and important unknown confounders may not be considered. Such bias may include (for example) that after serious accidents, participants may have an impaired mobility and consequently higher risk for cardiovascular risk factors and MI. Another example is that the death of a spouse may suggest poorer health of the participants themselves. The results are based solely on self-report. The prevalence of obesity, hypertension, and diabetes in our sample is lower compared to other population-representative data [81,82,83]. The representativeness of the sample is limited to age and gender of the German population. Besides self-reporting, there may be other significant factors that may cause the seen differences in prevalence. Although analyses were controlled for symptoms of anxiety and depression, the used tools are screening instruments and are not adequate to exclude a confounding effect of mental health problems. Although we have controlled for a number of covariates, other factors may drive the shown associations such as sleeping problems, drug abuse, risky behavior, social problems and more. CM may be affected by underreporting due to recall biases, denial, embarrassment and misunderstanding, which may impact the results [58,84]. Important characteristics such as timing and chronicity of maltreatment were not assessed. Nevertheless, the presented data give a meaningful insight into the relevance of the contribution of CM and stressful life events in adulthood for cardiovascular health.

## 5. Conclusions

The results of our study demonstrate that the experience of CM and stressful life events in adulthood are significantly associated with cardiovascular health of women and men in the German population. Cardiologists, general practitioners, and policymakers should be aware of the association between psychosocial stressors in childhood and adulthood with cardiovascular health.

The association of adult stressful life events with cardiovascular health was not moderated by CM, but CM and stressful life events predicted cardiovascular risk independent of each other in a dose-dependent manner. This finding highlights the relevance of stressors across the lifespan for cardiovascular health. While death of close persons and accidents are hard to avoid, public health strategies should focus on prevention of interpersonal violence before and after the age of 18 to reduce the burden of cardiovascular problems in the general population. Further longitudinal studies including measures of biological stress responses and potential confounding factors such as mental health, and behavioral and socioeconomic aspects are needed to improve our understanding of the importance of early life stress and stress in adulthood for cardiovascular health.

## Figures and Tables

**Table 1:** Sample characteristics. Presented as number of participants (%) unless otherwise stated. Gender differences were assessed with Chi^2^-tests/T-tests.

	Women 1339 (53.3)	Men 1171 (46.7)	Total 2510
Age, Mean (SD)	48.9 (18.1)	47.8 (18.4)	48.4 (18.2)
High educational level (baccalaureate) (%)	270 (20.3)	273 (23.4)	543 (21.6)
Risk of alcohol abuse (%)	94 (7.0)	204 (17.4)	298 (11.9)
Smoking (%)	389 (29.2)	452 (38.9)	842 (33.7)
Relevant symptoms of anxiety (%)	119 (8.9)	86 (5.8)	187 (7.5)
Relevant symptoms of depression (%)	116 (8.7)	66 (5.7)	182 (7.3)
Stressful life events in adulthood			
Loss of a partner or child	207 (15.8)	77 (6.7)	284 (11.6)
Severe accident	135 (10.2)	215 (18.6)	350 (14.1)
Sexual abuse after the age of 18	19 (1.5)	1 (0.1)	20 (0.8)
Workplace mobbing	91 (7.0)	45 (3.9)	136 (5.5)
Ever experienced any form of stressful life event (%)	380 (28.5)	298 (25.6)	678 (27.2)
Child maltreatment (CTQ)			
Emotional abuse (%)	115 (8.7)	48 (4.1)	163 (6.5)
Physical abuse (%)	86 (6.5)	81 (6.9)	167 (6.7)
Sexual abuse (%)	150 (11.3)	40 (3.4)	190 (7.6)
Emotional neglect (%)	168 (12.9)	149 (12.9)	317 (12.9)
Physical neglect (%)	286 (21.5)	267 (22.9)	553 (22.1)
Experienced any form of child maltreatment (%)	414 (31.9)	330 (28.6)	744 (30.3)
Cardiovascular risk factors or conditions			
Obesity (%)	259 (19.5)	157 (13.5)	416 (16.7)
Diabetes (%)	85 (6.4)	79 (6.8)	164 (6.6)
Hypertension (%)	315 (23.8)	264 (22.7)	579 (23.3)
Myocardial Infarction (%)	14 (1.1)	45 (3.9)	59 (2.4)

**Table 2:** Predictors of cardiovascular risk factors and myocardial infarction. Stratified for gender, analyzed via logistic regression analysis.

	Women			Men		
	OR	95% CI	*p*-Value	OR	95% CI	*p*-Value
**Obesity**						
Adult stressful life events	1.61	[1.27; 2.04]	<0.001	1.43	[1.06; 1.93]	0.019
Age	1.00	[0.99; 1.01]	0.651	1.01	[1.00; 1.02]	0.023
Education	1.07	[0.75; 1.51]	0.723	0.77	[0.52; 1.14]	0.195
Risk alcohol abuse	1.24	[0.71; 2.16]	0.453	0.42	[0.28; 0.62]	<0.001
Smoking	0.70	[0.52; 0.95]		1.04	[0.72; 1.51]	0.817
Chi^2^ (df)	20.41.34 (5)			38.37 (5)		
R^2^	0.02			0.06		
**Diabetes**						
Adult stressful life events	1.52	[1.09; 2.13]	0.014	1.54	[1.05; 2.27]	0.029
Age	1.05	[1.03; 1.07]	< 0.001	1.05	[1.04; 1.07]	<0.001
Education	1.34	[0.67; 2.71]	0.410	0.76	[0.43; 1.36]	0.357
Risk alcohol abuse	0.63	[0.33; 1.96]	0.630	0.60	[0.33; 1.10]	0.099
Smoking	0.94	[0.53; 1.68]	0.844	1.74	[0.99; 3.05]	0.054
Chi^2^ (df)	69.04 (5)			72.50 (5)		
R^2^	0.14			0.16		
**Hypertension**						
Adult stressful life events	1.23	[0.98; 1.54]	0.075	1.19	[0.91; 1.56]	0.207
Age	1.06	[1.05; 1.07]	<0.001	1.06	[1.06; 1.07]	<0.001
Education	1.47	[0.99; 2.20]	0.059	1.02	[0.70; 1.48]	0.917
Risk alcohol abuse	0.81	[0.47; 1.40]	0.442	0.65	[0.44; 0.915]	0.025
Smoking	1.19	[0.85; 1.67]	0.307	0.91	[0.66; 1.26]	0.572
Chi^2^ (df)	243.31 (5)			186.45 (5)		
R^2^	0.25			0.23		
**Myocardial infarction**						
Adult stressful life events	2.10	[1.03; 4.28]	0.040	1.76	[1.10; 2.82]	0.019
Age	1.06	[1.01; 1.10]	0.012	1.063	[1.04; 1.09]	< 0.001
Education	2.36	[0.29; 19.02]	0.419	2.25	[0.78; 6.50]	0.136
Risk alcohol abuse	0.31	[0.06; 1.53]	0.151	0.69	[0.31; 1.50]	0.344
Smoking	0.74	[0.19; 3.19]	0.736	1.23	[0.61; 2.46]	0.565
Chi^2^ (df)	20.58 (5)			62.65 (5)		
R^2^	0.14			0.19		

OR = Odds Ratio, 95% CI = 95 Confidence Interval.

**Table 3:** Predictors of cardiovascular risk factors and myocardial infarction. Stratified analyzed via stepwise logistic regression.

		Obesity			Diabetes			Hypertension			Myocardial Infarction	
	OR	95% CI	*p*-Value	OR	95% CI	*p*-Value	OR	95% CI	*p*-Value	OR	95% CI	*p*-Value
**Model 1**												
Gender	1.64	[1.30; 2.07]	<0.001	0.90	[0.63; 1.28]	0.550	1.00	[0.81; 1.24]	0.996	0.22	[0.12; 0.43]	<0.001
Age	1.00	[1.00; 1.01]	0.180	1.05	[1.04; 1.06]	<0.001	1.06	[1.05; 1.07]	<0.001	1.06	[1.04; 1.08]	<0.001
Education	0.86	[0.66; 1.13]	0.288	0.94	[0.59; 1.48]	0.781	1.17	[0.89; 1.55]	0.253	1.97	[0.76; 5.10]	0.162
Risk alcohol abuse	0.71	[0.51; 0.98]	0.039	0.82	[0.48; 1.38]	0.448	0.74	[0.54; 1.02]	0.068	0.87	[0.40; 1.91]	0.730
Smoking	0.86	[0.68; 1.09]	0.217	1.36	[0.90; 2.06]	0.145	1.05	[0.83; 1.33]	0.674	1.29	[0.67; 2.49]	0.444
Symptoms of anxiety	2.72	[1.72; 4.29]	<0.001	2.22	[1.13; 4.37]	0.020	1.22	[0.75; 1.98]	0.429	2.13	[0.76; 5.99]	0.152
Depressive symptoms	1.02	[0.63; 1.65]	0.939	1.02	[0.49; 2.12]	0.957	1.65	[1.02; 2.66]	0.042	1.36	[0.45; 4.12]	0.588
Adult stressful life events	1.39	[1.14; 1.68]	0.001	1.40	[1.07; 1.82]	0.013	1.15	[0.96; 1.37]	0.132	1.85	[1.23; 2.78]	0.003
Chi^2^ (df)			83.91 (8)			132.89 (8)		411.95 (8)				101.29 (8)
R^2^			0.06			0.14		0.24				0.21
**Model 2**												
Gender	1.63	[1.29; 2.05]	<0.001	0.88	[0.62; 1.26]	0.485	0.99	[0.80; 1.23]	0.963	0.20	[0.10; 0.40]	<0.001
Age	1.00	[1.00; 1.01]	0.230	1.05	[1.04; 1.06]	<0.001	1.06	[1.05; 1.07]	<0.001	1.06	[1.04; 1.08]	<0.001
Education	0.84	[0.64; 1.10]	0.215	0.89	[0.56; 1.41]	0.623	1.14	[0.86; 1.50]	0.369	1.79	[0.69; 4.66]	0.233
Risk alcohol abuse	0.72	[0.52; 0.99]	0.046	0.85	[0.50; 1.43]	0.535	0.76	[0.55; 1.05]	0.093	0.95	[0.43; 2.12]	0.909
Smoking	0.87	[0.69; 1.11]	0.271	1.41	[0.93; 2.14]	0.109	1.07	[0.84; 1.35]	0.589	1.41	[0.73; 2.75]	0.310
Symptoms of anxiety	2.59	[1.63; 4.09]	<0.001	1.93	[0.98; 3.80]	0.059	1.14	[0.70; 1.87]	0.592	1.52	[0.54; 4.29]	0.424
Depressive symptoms	0.97	[0.60; 1.56]	0.885	0.96	[0.47; 1.97]	0.909	1.52	[0.93; 2.46]	0.092	1.38	[0.48; 4.00]	0.549
Adult stressful life events	1.37	[1.13; 1.66]	0.002	1.37	[1.05; 1.78]	0.019	1.12	[0.94; 1.34]	0.202	1.84	[1.23; 2.75]	0.003
Childhood maltreatment	1.11	[1.00; 1.23]	0.041	1.21	[1.05; 1.40]	0.008	1.15	[1.05; 1.27]	0.005	1.38	[1.11; 1.71]	0.004
Chi^2^ (df)			87.95 (9)			139.40 (9)			419.84 (9)			108.91 (9)
R^2^			0.06			0.15			0.24			0.23

## Data Availability

The data presented in this study are available on request from the corresponding author. The data are not publicly available due to missing informed consent for public data sharing.

## Supplementary Materials

The following are available online at https://www.mdpi.com/article/10.3390/jcm10173937/s1. Table S1: Moderation analysis of adult stressful life events, child maltreatment and cardiovascular outcomes; Table S2: Predictors of cardiovascular risk factors and myocardial infarction, differentiated for interpersonal and other adult stressful life events as well as childhood abuse and childhood neglect. Analyzed via stepwise logistic regression, adjusted for gender and age.
