# Supplementary material for: The Interplay between Child Maltreatment and Stressful Life Events during Adulthood and Cardiovascular Problems—A Representative Study
Source: J Clin Med. 2021 Aug 31;10(17):3937. doi: 10.3390/jcm10173937 (PMC8432252; doi:10.3390/jcm10173937)
Supplement: Supplementary file 1 [file jcm-10-03937-s001.zip › jcm-1298728-supplementary.pdf]

Table S1. Moderation analysis of adult stressful life events, child maltreatment and cardiovascular outcomes.

	Chi² (df)	p-value	R^2	Z-value	95% CI	p-value
**Obesity**						
	41.20 (4)	<0.001	0.03			
Adult stressful life events				3.48	[0.17;0.62]	<0.001
Child maltreatment				2.58	[0.04;0.28]	0.010
Interaction				0.37	[-0.11; 0.16]	0.714
Age				0.37	[-0.01;0.01]	0.711
**Diabetes**						
	140.30 (4)	<0.001	0.15			
Adult stressful life events				2.31	[0.06;0.70]	0.021
Child maltreatment				2.75	[0.07;0.44]	0.006
Interaction				-0.21	[-0.20;0.16]	0.8326
Age				8.44	[0.04;0.06]	< 0.001
**Hypertension**						
	422.23(4)	<0.001	0.24			
Adult stressful life events				2.09	[0.01;0.43]	0.037
Child maltreatment				3.73	[0.11;0.34]	<0.001
Interaction				-0.97	[-0.20;0.07]	0.330
Age				16.17	[0.05;0.06]	<0.001
**MI**						

	85.25 (4)	0.711	0.17			
Adult stressful life events				2.22	[0.07;1.07]	0.026
Child maltreatment				2.74	[0.11;0.67]	0.006
Interaction				-0.50	[-0.31;0.19]	0.616
Age				5.99	[0.04;0.08]	<0.001

Table S2. Predictors of cardiovascular risk factors and myocardial infarction, differentiated for interpersonal and other adult stressful life events as well as childhood abuse and childhood neglect. Analyzed via stepwise logistic regression, adjusted for gender and age.

	Obesity			Diabetes			Hypertension			Myocardial infarction		
	**OR**	**95% CI**	**p-value**	**OR**	**95% CI**	**p-value**	**OR**	**95% CI**	**p-value**	**OR**	**95% CI**	**p-value**
**Model 1**												
Interpersonal adult stressful life events	2.54	1.80; 3.59	0.000	1.00	0.52; 1.92	0.997	1.53	1.06; 2.21	0.023	1.54	0.55; 4.28	0.408
Other adult stressful life events	1.29	1.03; 1.62	0.026	1.69	1.26; 2.26	<0.001	1.15	0.93; 1.40	0.190	2.04	1.33; 3.15	0.001
Chi² (df)	53.99 (4)			129.35 (4)			411.983 (4)			99.53 (4)		
R^2	0.037			0.138			0.24			0.20		
**Model 2**												
Interpersonal adult stressful life events	2.31	1.63; 3.29	0.000	0.84	0.43; 1.64	0.611	1.39	0.96; 2.02	0.082	1.32	0.47; 3.71	0.602

Other adult stressful life events	1.25	1.00; 1.58	0.054	1.63	1.21; 2.18	0.001	1.12	0.92; 1.38	0.266	1.98	1.28; 3.08	0.002
Childhood abuse	1.31	1.08; 1.58	0.006	1.45	1.11; 1.89	0.006	1.20	0.99; 1.46	0.067	1.44	0.98; 2.13	0.065
Childhood neglect	1.03	0.86; 1.24	0.748	1.15	0.89; 1.50	0.285	1.22	1.04; 1.44	0.018	1.52	1.03; 2.25	0.035
Chi² (df)	64.06 (6)			143.20 (6)			472.15 (6)			112.90 (6)		
R^2	0.044			0.15			0.25			0.23		
